# The Evaluation of the GET.ON Nationwide Web-Only Treatment Service for Depression- and Stress-Related Symptoms: Naturalistic Trial

**DOI:** 10.2196/42976

**Published:** 2024-02-01

**Authors:** Anne Etzelmueller, Elena Heber, Hanne Horvath, Anna Radkovsky, Dirk Lehr, David Daniel Ebert

**Affiliations:** 1 Department of Sports and Health Sciences, Professorship Psychology and Digital Mental Health Care, Technical University of Munich Munich Germany; 2 HelloBetter, GET.ON Institut für Online Gesundheitstrainings GmbH Hamburg / Berlin Germany; 3 Department of Clinical Psychology and Psychotherapy, Institute of Psychology, Friedrich-Alexander-University of Erlangen-Nürnberg Erlangen Germany; 4 Department of Psychology, Philipps University Marburg Marburg Germany; 5 Department of Health Psychology, Institute of Psychology, Leuphana University Lüneburg Lüneburg Germany

**Keywords:** depression, stress, digital, internet, effectiveness, routine care

## Abstract

**Background:**

GET.ON (HelloBetter) treatment interventions have been shown to be efficacious in multiple randomized controlled trials.

**Objective:**

This study evaluated the effectiveness of 2 GET.ON interventions, GET.ON Mood Enhancer and GET.ON Stress, in a national digital mental health service implemented across Germany.

**Methods:**

Following an initial web-based questionnaire, participants were allocated to either intervention based on their baseline symptom severity and personal choice and received a semistandardized guided, feedback-on-demand guided, or self-guided version of the treatment. Uncontrolled routine care data from 851 participants were analyzed using a pretest-posttest design. Half of the participants (461/851, 54.2%) were allocated to the stress intervention (189/461, 41% semistandardized; 240/461, 52% feedback on demand; and 32/461, 6.9% self-guided), and almost all participants in the mood intervention (349/352, 99.2%) received semistandardized guidance.

**Results:**

Results on depression-related symptom severity indicated a reduction in reported symptoms, with a large effect size of *d*=−0.92 (95% CI −1.21 to −0.63). Results on perceived stress and insomnia indicated a reduction in symptom severity, with large effect sizes of *d*=1.02 (95% CI −1.46 to −0.58) and *d*=−0.75 (95% CI −1.10 to −0.40), respectively. A small percentage of participants experienced deterioration in depression-related symptoms (11/289, 3.8%), perceived stress (6/296, 2%), and insomnia (5/252, 2%). After completing treatment, 51.9% (150/289) of participants showed a clinically reliable change in depression-related symptoms, whereas 20.4% (59/289) achieved a close to symptom-free status. Similar improvements were observed in perceived stress and insomnia severity. Guidance moderated the effectiveness of and adherence to the interventions in reducing depressive symptom severity. Effect sizes on depression-related symptom severity were *d*=−1.20 (95% CI −1.45 to −0.93) for the semistandardized group, *d*=−0.36 (95% CI −0.68 to −0.04) for the feedback-on-demand group, and *d*=−0.83 (95% CI −1.03 to −0.63) for the self-guided group. Furthermore, 47.6% (405/851) of the participants completed all modules of the intervention. Participant satisfaction was high across all patient groups and both interventions; 89.3% (242/271) of participants would recommend it to a friend in need of similar help. Limitations include the assignment to treatments and guidance formats based on symptom severity. Furthermore, part of the differences in symptom change between groups must be assumed to be due to this baseline difference in the measures.

**Conclusions:**

Future digital health implementation and routine care research should focus on monitoring symptom deterioration and other negative effects, as well as possible predictors of deterioration and the investigation of individual patient trajectories. In conclusion, this study supports the effectiveness of tailored digital mental health services in routine care for depression- and stress-related symptoms in Germany. The results highlight the importance of guidance in delivering internet-based cognitive behavioral therapy interventions and provide further evidence for its potential delivered as web-only solutions for increasing access to and use of psychological treatments.

## Introduction

### Background

Major depressive disorder is not only highly prevalent [1] but also associated with substantial impairment [2] and economic costs [3]. Although psychological interventions for depression have been shown to be effective [4], most individuals with depression fail to receive minimally adequate intervention [5]. In a large German sample (N=1186) reporting a 12-month diagnosis of a mental disorder, Mack et al [6] found that most individuals (81.1%) had not used mental health offers in the previous 12 months. Furthermore, they found a substantial time lapse of approximately 7 years between the onset of a mood disorder and service use.

Internet-based cognitive behavioral therapy (iCBT) may help overcome some of the limitations of traditional intervention services [7]. iCBT interventions have a broad reach and accessibility and might especially attract people who might not make use of traditional mental health services [8,9], whereas acceptability is reported to be lower in self-guided iCBT interventions than in other interventions [10]. With regard to the efficacy of iCBT, a recent meta-analysis has provided evidence that iCBT for depression can have positive effects on the mean symptom improvement for subthreshold depression [11] or major depressive disorder [12], resulting in clinically relevant changes in terms of response and remission [13], with effects found to be comparable with face-to-face psychotherapy [8]. In addition, recent research has shown that self-guided iCBT interventions result in significant effects on depression outcomes [14] as well, whereas guided interventions have yielded better outcomes in comparative reviews [15,16].

Recently, evidence for the effectiveness of guided iCBT interventions in routine mental health care has accumulated. Within a systematic literature review and meta-analysis, iCBT for the treatment of adult depression and anxiety has been shown to be effective when implemented in routine mental health care, with a within-group effect size of *g*=0.42-1.88 and a pooled effect of 1.18 for depression studies and 0.94 for anxiety studies [17]. In their review of computer-based interventions in randomized trials, Andrews et al [18] identified 8 studies on iCBT in routine clinical practice with an average within-group effect size of *g*=1.07, indicating symptom reduction across the treatment of depression, panic disorder, generalized anxiety disorder, and social phobia [18].

The GET.ON (today HelloBetter) Mood Enhancer is a German iCBT intervention for depression that has been evaluated in several randomized controlled trials (RCTs) with demonstrated efficacy in different target populations [19-23]. A study by Reins et al [21] found between-group effects for guided versions of the intervention of *d*=0.36 (95% CI 0.01-0.70; *P*=.03) in major depression when compared with web-based psychoeducation. In this study, both groups showed significant reductions in depression severity from pretreatment to posttreatment time points—8.31 points (*d*=1.09, 95% CI 0.72-1.46; *P*<.001) and 5.42 points (*d*=0.59, 95% CI 0.24-0.94; *P*<.001) on the Hamilton Rating Scale for Depression, respectively. Additional studies on treating depression-related symptoms in adults with type 1 and type 2 diabetes indicate the effectiveness of the adaptation of this intervention, with effects of *d*=0.58 at posttreatment measurement and *d*=0.83 (95% CI 0.57-1.08) after 6 months for depression and diabetes compared with web-based psychoeducation [19,20]. Moderator analyses also indicated the effectiveness in nonsuicidal participants with severe depression, with *d*=1.05 (95% CI 0.11-1.98) at posttreatment measurement and *d*=0.71 (95% CI 0.19-1.61) at the 6-month follow-up [24]. In addition, studies have found between-group effects for guided versions of the intervention of *d*=0.69 (95% CI 0.49-0.89) in subclinical depression compared with psychoeducation [22] and treatment as usual and *d*=0.37 (95% CI 0.09-0.64) when delivered with feedback-on-demand guidance only [23]. The intervention has also been found to be effective in reducing the risk of onset or delaying the onset of a major depressive disorder for 12 months [25]. The results of health economic studies showed that the guided intervention for depression and diabetes had a 97% probability of being cost-effective (at a willingness-to-pay ceiling of €5000 [US $5619.25] for a treatment response) compared with an active control group [26] and that the guided stress intervention indicated a 67% likelihood of being more cost-effective than no immediate intervention, whereas it showed net savings of €181 [US $203.42] on average per participant already in the first 6 months following the intervention [27].

Targeting individuals experiencing mild to moderate depression- and stress-related symptoms with interventions directed at stress might be a possibility to reach those individuals who are waiting for a specialized mental health treatment or might usually not seek help [9,28]. As individuals with mental health problems wait on average 3 months on an outpatient psychotherapeutic treatment [29], offering low-threshold interventions can help bridge the time until another evidence-based treatment is available. Reasons for the limited health care use also include the preference for solving problems on one’s own [30]. iCBT interventions might attract this population as they allow for independent and self-reliant work processes. In addition, stigmatizing attitudes toward mental illness are associated with less active help seeking for mental health problems [31]. Targeting mental health issues with a stress intervention might appeal to individuals potentially not seeking help otherwise, for example, providing interventions specifically labeled as targeting depression. Studies have shown that internet-based cognitive behavioral stress and occupational health interventions targeting depression can be effective [32,33]. Such interventions include techniques based on cognitive behavioral therapy principles, which have been shown to be effective in treating depression [34,35]. Weisel et al [36] showed that the GET.ON (today HelloBetter) internet-based stress management intervention is also effective for overcoming depressive symptoms and that it poses an adequate way for even highly affected participants to make use of psychotherapeutic interventions [36]. Effect sizes were moderate to large compared with the waitlist control condition both at the postassessment time point (*d*=0.67, 95% CI 0.32-1.02) and 6-month follow-up (*d*=0.79, 95% CI 0.44-1.15). In addition, in this study, 86.5% of participants reported having no experience with health-related digital applications, and 60.7% reported not having had experiences with face-to-face psychotherapy. These results indicate that such interventions might be a good entry point for individuals already showing high levels of depression who otherwise might not seek treatment.

The iCBT intervention GET.ON Stress, a web-based iCBT intervention including problem-solving and emotion regulation techniques, has been evaluated in 7 RCTs so far, demonstrating efficacies within different target groups [32,37,38]. In samples of employees with elevated symptoms of perceived stress, these studies found between-group effects for the self-guided intervention of *d*=0.83 (95% CI 0.58-1.08) at posttest measurement and *d*=1.02 (95% CI 0.76-1.27 [37]) at follow-up compared with waitlist controls and *d*=0.79 (95% CI 0.54-1.04) at posttest measurement and *d*=0.85 (95% CI 0.59-1.10 [32]) at follow-up for the intervention including feedback-on-demand guidance. Similarly, in college students reporting elevated symptoms of perceived stress, Harrer et al [39] found between-group effects for the feedback-on-demand guided intervention of *d*=0.69 (95% CI 0.36-1.02) at posttreatment measurement and *d*=0.57 (95% CI 0.24-0.89) at the 6-month follow-up compared with a waitlist control.

Taken together, there is evidence suggesting that guided and unguided self-help interventions for the treatment of depression are effective in a research setting within RCTs. However, the effectiveness outside of a highly structured research setting is less documented. In comparison with reports on RCTs, these often uncontrolled studies might present higher generalizability [40], whereas RCTs trade external validity for maximal internal validity [41,42]. Furthermore, RCTs often apply stricter inclusion and exclusion criteria than studies administered under routine care conditions and provide a highly structured setting accompanying the intervention setting [43]. Rothwell [41] found that the proportion of individuals with a specific disorder in a specific area recruiting for a trial would often be <1%. In addition, RCTs are assumed to have a potential adherence-fostering effect because of their highly structured nature [43,44]. Thus, the efficacy derived from RCTs of internet-based interventions might be overestimated for what can be expected when implementing them in routine care, limiting the knowledge base for routine practice [42]. Reporting on the effectiveness of interventions after establishing their efficacy in a controlled setting is important as efficacy trials may or may not yield similar effects in routine care conditions [45], and establishing the effectiveness of an intervention to evaluate its safety and scalability is crucial. Nonrandomized trials and open cohort studies on pretest-posttest (within-group) effects investigate events in a natural setting without the involvement of experimental interference.

In several recent effectiveness studies, a greater focus has been put on factors such as the quality of iCBT interventions and their clinical outcomes when delivered under naturalistic conditions [17]. These studies reported the within-group effect sizes of guided iCBT interventions (Hedges *g*=0.42−1.88, with a pooled effect of *g*=1.78 for depression treatments [17]).

Following this evidence, in 2015, the GET.ON Mood Enhancer and GET.ON Stress were implemented in German routine health care. Interventions provided by GET.ON are aimed at adults experiencing depression- and stress-related symptoms within a nationwide, web-only service for the prevention of and early intervention on depression- and stress-related symptoms. Clients were provided with a brief online assessment followed by semistandardized, feedback-on-demand, or self-guided iCBT.

### Objectives

The aim of this study was to investigate the use of, adherence to, effectiveness of, and patient satisfaction with this semistandardized, feedback-on-demand, and self-guided iCBT intervention for the treatment of depression- and stress-related symptoms when initially implemented in routine care. Participants were recommended either a stress- or depression-related intervention in a guided or self-guided format based on their baseline depression- and stress-related symptom severity. Using this tailored approach allowed for a broader implementation of the service and a higher reach in comparison with a *one-size-fits-some* approach.

## Methods

### Design

This effectiveness study used data routinely collected between January 2015 and June 2017 within a service provided in partnership with one of Germany’s major health insurance companies (HICs). All service users were invited to participate in this open trial using a naturalistic within-group design. We included all participants in the study who reported a Patient Health Questionnaire–8 (PHQ-8) [46,47] score of >5 at baseline and started the intervention. This study reports on GET.ON clients treated during the course of the MasterMind project [48], a European project set up to foster iCBT uptake in Europe. Today, the service is offered by HelloBetter within the German health care system and internationally.

### Procedure

The GET.ON (now HelloBetter) service comprised a fully web-based service without face-to-face client-coach interaction. Participants became aware of the intervention via the cooperating HIC’s personal recommendation or their health insurance online platform (members’ portal) or members’ magazine. Furthermore, clients were informed on the GET.ON website and the general media. Participants interested in the service registered either via the HIC’s or GET.ON’s system and could then access the intervention platform. There, the client filled out the screening questionnaire. The sole inclusion criteria for participation were being insured with the cooperating HIC and being aged >18 years. No further screening took place, and no clients were excluded with regard to any other criteria. Clients participated in the intervention anonymously. Upon entering the intervention platform, clients provided information on the intake questionnaires, namely, PHQ-8 [46,47,49], Perceived Stress Scale (PSS-10) [50,51], and Insomnia Severity Index (ISI) [52]. On the basis of the results, a psychologist provided the client with their individual intervention recommendation within 24 hours. The recommendation was based on the combined consideration of depression-related symptoms and perceived stress—a high PHQ-8 indicating higher levels of depression-related symptoms led to the recommendation of GET.ON Mood Enhancer even if perceived stress was elevated as well. Lower levels of depression-related symptoms combined with higher levels of perceived stress resulted in the recommendation of GET.ON Stress. Clients with high ISI values were recommended additional content focusing on sleep (sleep hygiene, stimulus control to enhance sleep quality, and the reduction of insomnia symptoms) in addition to the regular iCBT intervention. The client then received access to their training modules.

### Ethical Considerations

Data were routinely collected within the treatment (mental health check) as part of the provision of the intervention within a partnership with one of Germany’s major HICs. The study was a retrospective participant data analysis using anonymized routine data for which it is not possible to trace the data back to individual participants. In accordance with German legal regulations (§ 15 MBO-Ä), which state that “physicians who participate in a research project which invades the mental or physical integrity of a human being, or uses human body material or data which can be traced to a particular individual, must ensure that advice on questions of professional ethics and professional conduct associated with the project is obtained from an Ethics Committee established at the responsible Chamber of Physicians, or from another independent, interdisciplinary Ethics Committee set up according to state law, before conducting the research” [53], ethics approval was not regarded as a requirement at the time the study was conducted, as the study solely included anonymized routine data. There was no compensation to the participants for either receiving the treatment or filling out the mental health checks.

### Intervention

The service consisted of the GET.ON Mood Enhancer and GET.ON Stress interventions, with the level of guidance tailored to both individual symptom severity and the participants’ preference.

GET.ON Mood Enhancer was an evidence-based internet-based intervention consisting of 6 modules (online lessons) and was mainly based on problem-solving and behavioral activation. The modules relied on evidence-based face-to-face manuals that have been shown to be effective at reducing depressive symptomatology, including psychoeducation, and exercises for behavioral activation, problem-solving, and relapse prevention, with 6 optional modules (sleep problems, time management, better sleep, antidepressive medication, relaxation techniques, and worrying) that could be chosen depending on the individual user needs or preferences. A strong emphasis was placed on homework assignments designed to integrate acquired coping skills into daily life. Relative to the standard version of the intervention, which was originally developed to target subclinical depressive symptoms, the current version was shortened, updated with regard to design, and simplified to also account for potentially reduced ability to concentrate among individuals with more severe depressive symptoms, including reducing the length of explanatory text.

The GET.ON Stress intervention was based on the transactional model of stress by Lazarus et al [54]. This intervention included both problem-solving and emotion regulation strategies. Important health behavior change principles such as goal setting, action planning, and coping planning were followed. GET.ON Stress consisted of 7 sessions and a booster session provided 4 weeks after training completion. Following psychoeducation (session 1), the participants learned a 6-step procedure to systematically solve problems (sessions 2-3). In sessions 4 to 6, the participants were introduced to emotion regulation techniques (muscle and breathing relaxation, acceptance of negative emotions, and self-support in difficult situations). Session 7 included planning for the future, in which participants set goals to maintain the achieved results. Moreover, the same 6 aforementioned optional modules were included in this version as well. The application of exercises in daily life was strongly recommended. The participants were advised to complete 1 to 2 sessions per week. The program included exercises, audio and video files, and downloadable material and was presented on a secured web-based platform. A more detailed description of the overall intervention can be found in the protocol of the accompanying efficacy trial [55], whereas the implemented version of the GET.ON Stress intervention did not include mobile coaching via SMS text messaging.

Standard operating procedures in case of a crisis, such as suicidal ideation and suicidality, included coaches contacting their supervisor. In the case of suicidal ideation, coaches sent a message to the participant taking up what the participant had been writing. In this message, information on suicidal ideation was provided, and further support options were laid out. In an attachment, a detailed description of further support options was sent to the participant, detailing intermediate-term (therapeutic options such as a general practitioner and face-to-face psychotherapy and how to schedule an appointment, social consulting options, and other consulting centers) and instant offers (crisis and emergency numbers). Furthermore, participants showing symptom deterioration were referred to more intensive care.

### Guidance and Professional Training

On the basis of the participants’ baseline depressive symptom severity (measured using the PHQ-8) and perceived stress (measured using the PSS-10), they were recommended a semistandardized, feedback-on-demand, or self-guided version of the intervention. Following their personal recommendation, participants could decide to enter the version with less guidance (from semistandardized to feedback on demand or from feedback on demand to self-guided), but they could not enter a more intensive guidance format (from self-guided to feedback on demand or from feedback on demand to semistandardized guidance).

The self-guided version was completely unguided and self-administered by the participant. For technical questions, participants were able to contact the IT team. The feedback-on-demand guidance included standardized reminders from the platform once a week. In addition, participants were able to ask questions or request feedback from their online coach at any time during the first 3 months. Within the semistandardized guidance, the coaches provided personalized written feedback based on templates after the completed treatment modules as a patient safety measure. Online coaches were licensed clinical psychologists or clinical psychologists in training for the license; in exceptional cases, they were psychologists under the supervision of experienced licensed clinical psychologists. Professional training included the provision of a detailed intervention manual and close supervision by an experienced licensed psychologist.

### Measures

All questionnaires were administered at baseline before starting the intervention as part of the intervention; afterward, the participants could fill in the questionnaires every 2 weeks. The analysis used postmeasurement data that were collected closest in time to the completion of the last treatment session that the participant engaged in.

#### Primary Outcome Measure

The PHQ-8 is a self-report measure for the assessment of depressive symptoms. It administers the first 8 items of the Patient Health Questionnaire–9 (PHQ-9) [46,47], omitting the item on thoughts of death or self-harm. The PHQ-8 has been used in clinical or research settings where the follow-up to positive responses to the ninth item of the PHQ-9 may be delayed, for example, in a web-based screening. Erbe et al [56] found that delivering the PHQ-9 in a digital format does not affect the psychometric properties in a clinically meaningful way.

#### Secondary Outcome Measures

As a secondary outcome, the level of perceived stress was measured using the PSS-10 (5-point Likert scale; range 0-40; Cronbach α=.78-.91 [50,51,57]). Furthermore, insomnia severity was measured using the ISI (7 items; range 0-28; Cronbach α=.90; [52]). The participants’ satisfaction was assessed using the Client Satisfaction Questionnaire–8 (CSQ-8; 8 items; range 0-32 [58,59]). The questionnaire has been translated into various languages and is used to measure global participant satisfaction.

### Statistical Analyses

Participant characteristics were analyzed using descriptive statistics. Linear mixed-effects models were applied to estimate intervention effects. We used Satterthwaite approximations [60] to derive *P* values for the fixed effects and calculated the effect sizes by dividing the estimated mean difference by the SD of the postmeasure [61]. We included the variables symptom severity (mild, moderate, and severe), guidance (semistandardized guided, feedback-on-demand guided, and self-guided), and intervention (mood and stress) into the linear models and compared this model with the model including the interaction of the variable with the measurement time point using chi-square tests. If a significant interaction was found, we investigated the effects of a linear mixed model for each level of the variable. As we applied 3 group comparisons, we adjusted the *P* value indicating significance to .05/3=.02 [62].

Intervention completers were defined as participants who started all the intended modules. A minimal adequate dose of the intervention was defined as starting at least 5 modules of any intervention. As the aforementioned statistical model assumes missing-at-random data, we also included the analysis using only completer data. To determine the number of participants achieving a reliable, positive outcome, we coded participants as responders or nonresponders according to the widely used Reliable Change Index [63]. To determine the potential negative effects of the intervention, the number of participants showing a reliable symptom deterioration was assessed regarding depressive symptom severity and perceived stress, defined as a negative change in symptom severity based on a negative Reliable Change Index. According to Jacobson and Truax [63], a cutoff point indicating symptom-free status was calculated and defined as scoring >2 SDs below the baseline mean.

Logistic regression analysis was conducted to explore the influence of depressive symptom severity (PHQ-8) at baseline, before the start of the intervention, and at completion. We also tested the influence of guidance (semistandardized, feedback on demand, and self-guided) on adherence (defined as the number of completed modules).

Participant satisfaction measured using the CSQ-8 was reported as means and SDs at the item level and as the “percentage of agreement” operationalized as the positive answer on the 4-point Likert scale on the CSQ-8. We analyzed the data of participants who filled out the complete questionnaire.

Information on participants’ recruitment pathways was provided in open-text answers. These text answers were interpreted and assigned to one of six groups: (1) information from the HIC, (2) via the internet (through the GET.ON, HIC website, membership portal, or Google), (3) from the HIC’s membership magazine, (4) through direct HIC consultation, (5) recommendations by health care professionals, or (6) recommendations by others (family and friends).

R (version 3.5.2; R Foundation for Statistical Computing) [64] was used for all analyses.

## Results

### Sample Selection and Baseline Characteristics

**Figure 1:**
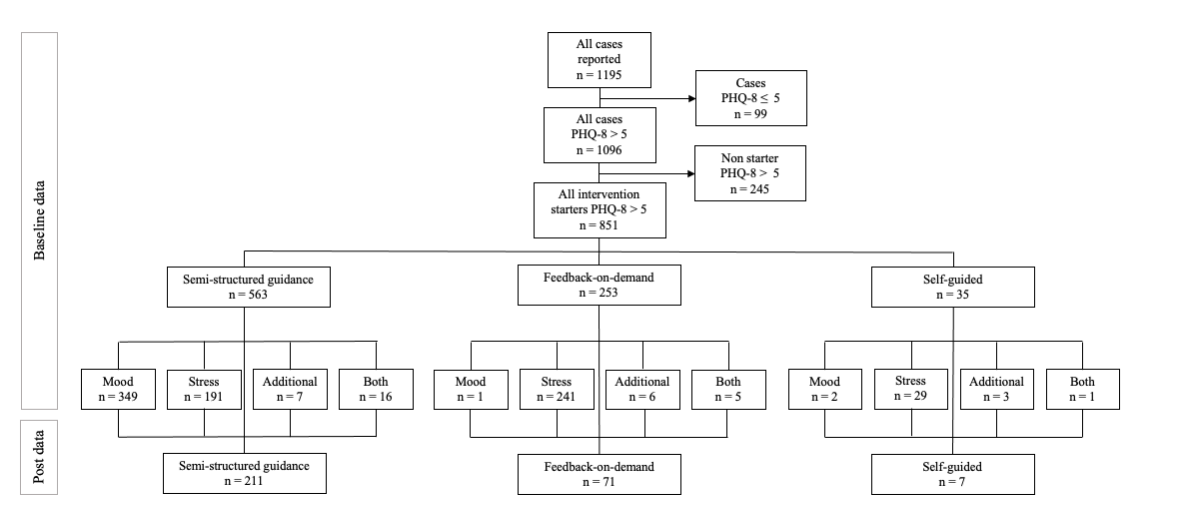
Participant flowchart. PHQ-8: Patient Health Questionnaire–8.

Figure 1 shows the participant flowchart. Of the 1195 cases reported in the data set, 1096 (91.72%) reported a PHQ-8 score of >5 at baseline. Of those 1096 participants, 851 (77.65%) also started the intervention (opened at least one session). Of those 851 participants, 327 (38.4%) were identified as having severe depressive symptoms according to the PHQ-8 and 310 (36.4%) were identified as having moderately severe symptoms. On average, participants were aged 41.7 (SD 11.31) years, and 72.2% (612/848) were female. Approximately half (405/833, 48.7%) were working full time, whereas 24.6% (205/833) were employed part time, 10.7% (89/833) were employed but on sick leave, and 7.2% (60/833) were unemployed. In total, 59.7% (503/843) had received a higher education, and 3.8% (32/843) had received a lower education. Of the included participants, 40.4% (337/834) reported having received psychotherapeutic treatment before, 18.3% (153/834) were in psychotherapeutic treatment while receiving the iCBT intervention, and 41.3% (345/834) had not received any form of psychotherapy before. Of the included participants, 38.4% (327/851) reported severe depressive symptoms, 36.4% (310/851) reported moderate depressive symptoms, and 25.1% (214/851) reported mild depressive symptoms at baseline. In addition, 98.7% (840/851) of participants followed the recommendation regarding the guidance format based on their symptom severity. Furthermore, 41.4% (352/851) of participants (*mild symptoms*: 1/352, 0.3%; *moderate symptoms*: 36/352, 10.2%; *severe symptoms*: 315/352, 89.5%) entered the mood intervention, 54.2% (461/851) of participants (*mild symptoms*: 196/461, 42.5%; *moderate symptoms*: 236/461, 51.2%; *severe symptoms*: 2/461, 0.4%) entered the stress management intervention, 2.6% (22/851) of participants (*mild symptoms*: 8/22, 36%; *moderate symptoms*: 8/22, 36%; *severe symptoms*: 6/22, 27%) entered both, and 1.9% (16/851) of participants started optional online modules only. Furthermore, 66.2% (563/851) of clients participated in the semistandardized guided intervention (*mood*: 349/563, 62%; *stress*: 191/563, 33.9%; *only optional modules*: 7/563, 1.2%; *both stress and depression*: 16/563, 2.8%), 29.7% (253/851) of clients participated in the feedback-on-demand guided intervention (*mood*: 1/253, 0.4%; *stress*: 241/253, 95.3%; *only optional modules*: 6/253, 2.4%; *both* *stress and depression*: 5/253, 2%), and 4.1% (35/851) of clients participated in the self-guided intervention (*mood*: 2/35, 6%; *stress*: 29/35, 83%; *only optional modules*: 3/35, 9%; *both stress and depression*: 1/35, 3%). Of all participants, 32.9% (279/851) were recruited via the internet, 23.3% (198/851) were recruited via direct contact with the HIC, and 19.9% (169/851) were recruited via the HIC’s membership magazine. In addition, 2.7% (23/851) were recruited via a direct HIC consultation, the treatment was recommended by their general practitioner to 0.5% (4/851), and the treatment was recommended by a nonprofessional (ie, friend or family) to 1.1% (9/851). Participants’ demographic characteristics are presented in Table 1.

**Table 1:** Demographics for all starters reporting a Patient Health Questionnaire–8 score of >5 (N=851).

Characteristics		Values
Age (years), mean (SD)		41.68 (11.31)
Sex (female), n (%)		612 (72.2)
**Employment, n (%)**		
	Employed but on sick leave	89 (10.7)
	Employed (part time)	205 (24.6)
	Employed (full time)	405 (48.7)
	Unemployed	60 (7.2)
	Nonworking (eg, pensioner, housewife, or househusband)	73 (8.8)
**Education, n (%)**		
	High	503 (59.7)
	Middle	306 (36.3)
	Low	32 (3.8)
	None	1 (0.1)
**Symptom severity, n (%)**		
	Mild	214 (25.1)
	Moderate	310 (36.4)
	Severe	327 (38.4)
**Experience with PT^a^, n (%)**		
	“I have received PT before”	337 (40.4)
	“I am currently in PT”	153 (18.3)
	“I have never received PT before”	345 (41.3)
**Intervention, n (%)**		
	Mood	352 (41.4)
	Stress	461 (54.2)
	Only optional modules	16 (1.9)
	Both mood and stress main modules	22 (2.6)
Number of sessions, mean (SD)		6.64 (4.85)
Number of sessions from the main intervention, mean (SD)		3.91 (2.64)
**Guidance modality, n (%)**		
	Feedback-on-demand guided	253 (29.7)
	Semistandardized guided	563 (66.2)
	Self-guided	35 (4.1)

^a^PT: psychotherapy.

### Primary Analysis: Effects on the Depression Measure

#### Statistical Significance and Effect Size

Table 2 shows the means and SDs of participants’ depressive symptom severity measured using the PHQ-8 at both measurement time points. The primary analysis showed a significant reduction in depressive symptoms on the PHQ-8 over time (β=−4.61; SE 0.27; *P*<.001), reflecting a large effect size of *d*=−0.92 (95% CI −1.21 to −0.63). Multimedia Appendix 1 depicts the sensitivity analyses for these outcomes and effect sizes for depressive symptom severity within the subsets of study and treatment completers.

**Table 2:** Means and SDs of participants’ Patient Health Questionnaire–8 (PHQ-8), Perceived Stress Scale (PSS-10), and Insomnia Severity Index (ISI) scores at both measurement time points.

			Participants, n (%)^a^		PHQ-8, mean (SD)		PSS-10, mean (SD)		ISI, mean (SD)
**All**									
	Before	851 (100)		13.02 (4.57)		25.77 (5.63)		13.65 (5.86)	
	After	307 (36.1)		8.65 (5.03)		18.23 (7.53)		9.37 (6.10)	
**Mood intervention**									
	Before	352 (41.3)		17.22 (2.87)		28.19 (5.43)		16.15 (5.64)	
	After	127 (14.9)		10.43 (5.46)		19.89 (7.49)		11.38 (6.44)	
**Stress intervention**									
	Before	461 (54.2)		9.94 (2.69)		24.06 (5.03)		11.79 (5.30)	
	After	148 (17.4)		7.07 (3.92)		16.68 (7.19)		7.63 (5.22)	
**Both interventions**									
	Before	22 (2.6)		12.05 (4.57)		25.52 (5.78)		12.68 (6.66)	
	After	13 (1.5)		9.46 (6.24)		19.92 (8.79)		9.90 (6.44)	
**Only optional modules**									
	Before	16 (1.8)		10.75 (5.08)		22.31 (6.59)		13.56 (5.14)	
	After	1 (0.1)		4.00 (N/A^b^)		17.00 (N/A)		6.00 (N/A)	
**Semistandardized guidance**									
	Before	563 (66.1)		15.51 (3.36)		27.87 (4.80)		14.98 (5.82)	
	After	211 (24.8)		9.36 (5.18)		19.00 (7.35)		9.80 (6.23)	
**Feedback-on-demand guidance**									
	Before	253 (29.8)		8.43 (1.41)		21.93 (4.77)		11.43 (4.90)	
	After	71 (8.3)		6.97 (4.14)		16.35 (7.91)		8.41 (5.72)	
**Self-guided**									
	Before	35 (4.1)		6.23 (3.61)		19.76 (4.99)		8.17 (4.99)	
	After	7 (0.8)		4.29 (0.95)		14.29 (4.82)		6.57 (4.35)	
**Mild symptoms**									
	Before	214 (25.1)		7.35 (1.42)		20.88 (4.73)		10.32 (4.88)	
	After	59 (6.9)		6.61 (4.08)		16.54 (8.16)		8.02 (5.36)	
**Moderate symptoms**									
	Before	310 (36.4)		11.87 (1.48)		25.44 (4.73)		12.88 (5.29)	
	After	106 (12.5)		7.78 (4.24)		17.17 (6.68)		7.72 (5.20)	
**Severe symptoms**									
	Before	327 (38.4)		17.83 (2.35)		29.30 (4.32)		16.55 (5.59)	
	After	124 (14.6)		10.35 (5.51)		19.96 (7.62)		11.39 (6.55)	

^a^Sample size in the subset was based on answers on the PHQ-8.

^b^N/A: not applicable.

#### Influence of Guidance on Treatment Outcome

Guidance was associated with depressive symptom severity over time (*F*_2,755.96_=45.53; *P*<.001). Planned comparisons of the guidance groups revealed that semistandardized guidance was associated with a greater symptom reduction compared with feedback-on-demand guidance (β=4.68; SD 0.51; *t*=9.23; *P*<.001) and the self-guided format (β=4.54; SD 1.38; *t*=3.28; *P*=.001). The effect of “self-guided” was not significant over time. Participants in the feedback-on-demand guided group did not show a significantly higher symptom reduction than those in the self-guided group (β=−.14; SD 1.43; *t*=−0.1; *P*=.92). The group sizes differed in this analysis as only 4.1% (35/851) of participants were included in the self-guided condition, 66.2% (563/851) were included in the semistandardized condition, and 29.7% (253/851) were included in the feedback-on-demand guided condition. The corresponding effect sizes in the guidance groups were *d*=−1.20 (95% CI −1.45 to −0.93) for the semistandardized guided group, *d*=−0.36 (95% CI −0.68 to −0.04) for the feedback-on-demand guided group, and *d*=−0.83 (95% CI −1.03 to −0.63) for the self-guided group.

#### Influence of the Intervention Type on Treatment Outcome

The intervention type (*mood* or *stress*) was associated with depression-related symptom severity measured using the PHQ-8 over time (*F*_1,680.77_=79.59; *P*<.001), with β=3.9 (SD 0.44; *t*=8.92; *P*<.001) indicating a higher symptom change in the mood treatment. The corresponding within-group effect sizes were *d*=−1.26 (95% CI −1.6 to −0.92) for the mood intervention and *d*=−0.75 (95% CI −0.97 to −0.53) for the stress intervention.

#### Influence of Baseline Symptom Severity on Treatment Outcome

Depression-related symptom severity was associated with intervention outcomes (*F*_2,858.05_=94.49; *P*<.001). Planned comparisons of depression-related symptom severity revealed that severe depression-related symptoms at baseline were associated with a greater symptom reduction compared with mild depression-related symptom severity (β=−6.64; SD 0.5; *t*=−13.42; *P*<.001), moderate depression-related symptoms were associated with a greater symptom reduction compared with mild depression-related symptom severity (β=−3.27; SD 0.51; *t*=−6.44; *P*<.001), and severe depression-related symptoms were associated with a greater symptom reduction compared with moderate depression-related symptom severity (β=3.37; SD 0.42; *t*=8.05; *P*<.001). The corresponding effect sizes in the symptom severity groups were *d*=−1.36 (95% CI −1.72 to −1.00) for severe depression-related symptoms, *d*=−1.00 (95% CI −1.26 to −0.68) for moderate depression-related symptoms, and *d*=−0.21 (95% CI −0.55 to 0.13) for mild depression-related symptoms. The results of the analyses of the pre- and postintervention changes in symptom severity are shown in Table 3.

**Table 3:** Linear mixed-effects model outcomes and effect sizes for depressive symptom severity (measured using the Patient Health Questionnaire–8).

			Estimate (SD)		*t* test		*F* test (*df*)		*P* value		Effect size (*d*) (95% CI)
**All starters (n=851)**							288.42 (504.90)		<.001		−0.92 (−1.21 to −0.63)
	Time	−4.61 (0.27)		−16.98							
**Guidance**							45.53 (755.96)		<.001		
	SSF^a^	−6.17 (0.28)		−21.70				<.001		−1.19 (−1.45 to −0.93)	
	FoD^b^	−1.47 (0.30)		−4.91				<.001		−0.36 (−0.68 to −0.04)	
	SG^c^	−0.76 (0.36)		−2.10				.08		−0.83 (−1.03 to −0.63)	
	SSF × SG	4.54 (1.38)		3.28				.001		N/A^d^	
	SSF × FoD	4.68 (0.51)		9.23				<.001		N/A	
	FoD × SG	−0.14 (1.43)		−0.10				.92		N/A	
**Intervention**							79.59 (680.77)		<.001		
	Mood	−6.87 (0.35)		−19.40				<.001		−1.26 (−1.60 to −0.92)	
	Stress	−2.95 (0.27)		−10.87				<.001		−0.75 (−0.97 to −0.53)	
**Symptom severity**							94.49 (858.05)		<.001		
	Severe	−7.48 (0.34)		−21.92				<.001		−1.36 (−1.72 to −1.00)	
	Medium	−4.09 (0.28)		−14.77				<.001		−0.97 (−1.26 to −0.68)	
	Mild	−0.85 (0.30)		−2.81				.005		−0.21 (−0.55 to −0.13)	
	Mild × severe	−6.64 (0.50)		−13.42				<.001		N/A	
	Mild × medium	−3.27 (0.51)		−6.44				<.001		N/A	
	Medium × severe	3.37 (0.42)		8.05				<.001		N/A	

^a^SSF: semistandardized feedback.

^b^FoD: feedback on demand.

^c^SG: self-guided.

^d^N/A not applicable.

#### Reliable Change

Table 2 shows the means and SDs of the participants’ PHQ-8 scores at both measurement time points. Of all participants reporting postmeasure data, 3.8% (11/289) experienced a deterioration in symptoms. In this study, the cutoff score for reliable change was 9.14, indicating a value of 2 SDs below the mean of the population at the premeasurement time point. At the postmeasurement time point, 51.9% (150/289) and 20.4% (59/289) of participants showed a clinically reliable change in depressive symptoms and close to symptom-free status, respectively. The reliable change, remission, and deterioration rates are shown in Table 4.

**Table 4:** Reliable change, deterioration, and remission^a^.

	Reliable change, n (%)	Deterioration, n (%)	Remission, n (%)
PHQ-8^b^	150 (51.9)	11 (3.80)	59 (20.4)
PSS-10^c^	152 (51.4)	6 (2)	109 (36.8)
ISI^d^	128 (50.8)	5 (2)	61 (24.2)

^a^Reliable Change Index was used following Jacobson and Truax [63]; a cutoff point indicating symptom-free status was calculated and defined as scoring >2 SDs below the mean (preintervention measurement).

^b^PHQ-8: Patient Health Questionnaire–8.

^c^PSS-10: Perceived Stress Scale.

^d^ISI: Insomnia Severity Index.

### Secondary Outcomes

Table 2 shows the means and SDs of participants’ perceived stress measured using the PSS-10 and insomnia severity measured using the ISI at both measurement time points.

#### Effects on Perceived Stress

##### Statistical Significance of Perceived Stress

There was a significant reduction in perceived stress on the PSS-10 over time (*F*_1,614.39_=404.73; *P<*.001), with β=−7.7 (SE 0.38; *P<*.001), reflecting a large effect size of *d*=−1.02 (95% CI −1.46 to −0.58). Results are depicted in Table 5. Multimedia Appendix 1 shows the sensitivity analyses for these outcomes and the effect sizes for perceived stress within the subsets of study and treatment completers.

**Table 5:** Linear mixed-effects model outcomes and effect sizes for perceived stress (measured using the Perceived Stress Scale).

		Estimate (SD)	*t* test	*F* test (*df*)		*P* value		Effect size (*d*; 95% CI)	
**All starters (n=847)**					404.73 (614.39)		<.001		−1.02 (−1.46 to −0.58)
	Time	−7.70 (0.38)	−20.12						
**Guidance**					8.38 (762.22)		<.001		
	SSF^a^	−8.88 (0.43)	−20.86			<.001		−1.21 (−1.58 to −0.84)	
	FoD^b^	−5.66 (0.70)	−8.06			<.001		−0.72 (−1.33 to −0.11)	
	SG^c^	−5.45 (1.67)	−3.26			.012		−1.18 (−2.18 to −0.18)	
	SSF × SG	3.42 (2.24)	1.52			.13		N/A^d^	
	SSF × FoD	3.24 (0.82)	3.93			<.001		N/A	
	FoD × SG	0.18 (2.31)	0.08			.94		N/A	
**Intervention**					1.15 (622.21)		.28		
	Mood	−8.35 (0.56)	−14.87			<.001		−1.12 (−1.59 to −0.65)	
	Stress	−7.49 (0.50)	−14.85			<.001		−1.04 (−1.44 to −1.44)	
**Symptom severity**					94.49 (858.05)		<.001		
	Severe	−9.30 (0.53)	−17.58			<.001		−1.22 (−1.72 to −0.72)	
	Medium	−8.30 (0.57)	−14.55			<.001		−1.25 (−1.70 to −0.80)	
	Mild	−4.57 (0.78)	−5.85			<.001		−0.56 (−1.24 to 0.12)	
	Mild × severe	−4.74 (0.93)	−5.12			<.001		N/A	
	Mild × medium	−3.75 (0.95)	−3.95			<.001		N/A	
	Medium × severe	0.99 (0.79)	1.26			.21		N/A	

^a^SSF: semistandardized feedback.

^b^FoD: feedback on demand.

^c^SG: self-guided.

^d^N/A: not applicable.

##### Influence of Guidance on Stress Treatment Outcome

Guidance was associated with the level of perceived stress (*F*_2,762,22_=8.38; *P*<.001). Planned comparisons of the guidance groups revealed that semistandardized guidance was associated with a greater symptom reduction compared with feedback-on-demand guidance (β=3.24; SD 0.82; *t*=3.93; *P*<.001) but not with self-guidance (β=3.42; SD 2.24; *t*=1.52; *P*=.13). Participants in the feedback-on-demand guided group did not show a significantly higher symptom reduction than those in the self-guided group (β=.18; SD 2.31; *t*=0.08; *P*=.94). The corresponding within-group effect sizes in the guidance groups were *d*=−1.21 (95% CI −1.58 to −0.84) for the semistandardized guided group, *d*=−0.72 (95% CI −1.33 to −0.11) for the feedback-on-demand guided group, and *d*=−1.18 (95% CI −2.18 to −0.18) for the self-guided group.

##### Influence of Treatment on Stress Treatment Outcome

There was no influence of the intervention (*mood* or *stress*) on the change in perceived stress over time (*F*_1,622.21_=1.15; *P=*.28), with β=.81 (SD 0.75; *t*=1.07; *P=*.28). The corresponding effect sizes were *d*=−1.12 (95% CI −1.59 to −0.65) for the mood intervention and *d*=−1.04 (95% CI −1.44 to −0.64) for the stress intervention.

##### Influence of Baseline Symptom Severity on Stress Treatment Outcome

Depression-related symptom severity was associated with perceived stress (*F*_2,745.27_=13.44; *P<*.001). Planned comparisons of the depression-related symptom severity groups revealed that severe depression-related symptoms at baseline were associated with a greater symptom reduction compared with mild symptom severity (β=−4.74; SD 0.93; *t*=−5.12; *P*<.001), and moderate depression-related symptoms led to a greater symptom reduction compared with mild depression-related symptom severity (β=−3.75; SD 0.95; *t*=−3.95; *P*<.001), whereas there was no significant difference between severe and moderate depression-related symptoms (β=.99; SD 0.79; *t*=1.26; *P*=.21). The corresponding effect sizes were *d*=−1.22 (95% CI −1.72 to −0.72) for severe depression-related symptoms, *d*=−1.25 (95% CI −1.7 to −0.8) for moderate depression-related symptoms, and *d*=−0.56 (95% CI −1.24 to 0.12) for mild depression-related symptoms.

##### Clinically Reliable Change in Perceived Stress

Furthermore, 2% (6/296) of the participants experienced a deterioration in symptoms. The cutoff score for clinically reliable change was 10.80, indicating a value of 2 SDs below the mean PSS-10 score of the population at the premeasurement time point. In total, 51.4% (152/296) of participants showed a clinically reliable change, and 36.8% (109/296) had a close to symptom-free status. The reliable change, remission, and deterioration rates are shown in Table 4.

#### Effects on Insomnia

##### Statistical Significance of Insomnia

There was a significant reduction in insomnia severity on the ISI over time (*F*_1,456.25_=192.32; *P*<.001), with β=−4.57 (SE 0.33; *P*<.001), reflecting a large effect size of *d*=−0.75 (95% CI −1.10 to −0.40). Results are depicted in Table 6.

**Table 6:** Linear mixed-effects model outcomes and effect sizes for insomnia severity (measured using the Insomnia Severity Index).

		Estimate (SD)	*t* test	*F* test (*df*)		*P* value		Effect size (*d*; 95% CI)	
**All starters (n=849)**					192.32 (456.25)		<.001		−0.75 (−1.10 to −0.40)
	Time	−4.57 (0.33)	−13.87						
**Guidance**					3.81 (523.37)		.023		
	SSF^a^	−5.21 (0.38)	−13.59			<.001		−0.84 (−1.15 to −0.53)	
	FoD^b^	−3.36 (0.63)	−5.33			<.001		−0.59 (−1.03 to −0.15)	
	SG^c^	−0.94 (0.87)	−1.08			.32		−0.22 (−1.11 to 0.67)	
	SSF × SG	3.93 (2.04)	1.93			.05		N/A^d^	
	SSF × FoD	1.59 (0.74)	2.15			.03		N/A	
	FoD × SG	2.34 (2.10)	1.12			.27		N/A	
**Intervention**					0.26 (461.31)		.61		
	Mood	−4.82 (0.49)	−9.81			<.001		−0.75 (−1.16 to −0.34)	
	Stress	−4.45 (0.43)	−10.39			<.001		−0.85 (−1.14 to −0.56)	
**Symptom severity**					94.49 (858.05)		<.001		
	Severe	−5.23 (0.50)	−10.36			<.001		−0.80 (−1.23 to −0.37)	
	Medium	−5.22 (0.53)	−9.83			<.001		−1.01 (−1.36 to −0.66)	
	Mild	−2.65 (0.64)	−4.15			<.001		−0.50 (−0.95 to −0.05)	
	Mild × severe	−2.59 (0.86)	−3.02			.003		N/A	
	Mild × medium	−2.61 (0.88)	−2.97			.003		N/A	
	Medium × severe	−0.02 (0.72)	−0.03			.97		N/A	

^a^SSF: semistandardized feedback.

^b^FoD: feedback on demand.

^c^SG: self-guided.

^d^N/A: not applicable.

##### Influence of Guidance on Insomnia Treatment Outcome

Guidance was associated with insomnia severity (*F*_2,523,37_=3.81; *P*=.02). Planned comparisons of the guidance groups revealed that there was a significant difference between semistandardized guidance and feedback-on-demand guidance (β=1.59; SD 0.74; *t*=2.15; *P*=.03) but no difference between semistandardized guidance and self-guidance (β=3.93; SD 2.04; *t*=1.93; *P*=.05) or between self-guidance and feedback-on-demand guidance (β=2.34; SD 2.1; *t*=1.12; *P*=.27). The corresponding effect sizes were *d*=−0.84 (95% CI −1.15 to −0.53) for semistandardized guidance, *d*=−0.59 (95% CI −1.03 to −0.15) for feedback-on-demand guidance, and *d*=−0.22 (95%-CI −1.11 to 0.67) for self-guidance.

##### Influence of the Type of Treatment on Insomnia Treatment Outcome

We did not find that the type of intervention (*mood* or *stress*) was associated with change in insomnia severity (*F*_1_=0.26; *P*=.61), with β=.33 (SD 0.65; *t*=0.51; *P*=.61). The corresponding effect sizes were *d*=−0.75 (95% CI −1.16 to −0.34) for the mood intervention and *d*=−0.85 (95% CI −1.14 to −0.56) for the stress intervention.

##### Influence of Baseline Symptom Severity on Insomnia Treatment Outcome

Depression-related symptom severity was associated with insomnia severity (*F*_2,517.42_=5.4; *P*<.001). Planned comparisons of the symptom severity groups revealed that severe depression-related symptoms at baseline were associated with a greater symptom reduction in insomnia compared with mild symptom severity (β=−2.59; SD 0.86; *t*=−3.02; *P*=.003), and moderate depression-related symptoms were associated with a greater symptom reduction compared with mild depression-related symptoms (β=−2.61; SD 0.88; *t*=−2.97; *P*=.003), whereas there was no significant difference between severe and moderate depression-related symptoms (β=−.02; SD 0.72; *t*=−0.03; *P*=.97). The corresponding effect sizes in the symptom severity groups were *d*=−0.80 (95% CI −1.23 to −0.37) for severe depression-related symptoms, *d*=−1.01 (95% CI −1.36 to −0.66) for moderate depression-related symptoms, and *d*=−0.50 (95% CI −0.95 to −0.05) for mild depression-related symptoms.

##### Clinically Reliable Change in Insomnia

Furthermore, 50.8% (128/252) of participants reporting insomnia severity scores at the postintervention measure reported a clinically reliable change in the insomnia measure (measured using the ISI) from baseline to postintervention measure, and 2% (5/252) experienced a deterioration in symptoms. In this study, the cutoff score was 11.73, indicating a value of 2 SDs below the mean ISI score of the population at the premeasurement time point. At the posttreatment time point, 24.2% (61/252) of participants showed a close to symptom-free status on the insomnia severity measure as defined by an ISI score of <8. The reliable change, remission, and deterioration rates are shown in Table 4. In total, 50.8% (128/252) of participants showed a clinically reliable change, and 24.2% (61/252) had a close to symptom-free status. The reliable change, remission, and deterioration rates are shown in Table 4.

#### Adherence

Table 7 reports the number and percentage of participants completing all intervention modules as intended as well as those receiving a minimal adequate dose of the intervention (at least 5 modules).

**Table 7:** Participant adherence.^a^

		Participants, n (%)	Completed all modules as intended, n (%)	Received a minimal adequate dose of the intervention (at least 5 modules), n (%)
All participants		851 (100)	405 (47.6)	474 (55.7)
**Per intervention**				
	Mood	352 (41.4)	184 (52.3)	210 (59.7)
	Stress	461 (54.2)	202 (43.8)	243 (52.7)
	Both	22 (2.6)	18 (81.8)	20 (90.9)
**Per guidance format**				
	Semistandardized	563 (66.2)	298 (52.9)	344 (61.1)
	Feedback on demand	253 (29.7)	98 (38.7)	120 (47.4)
	Self-guided	35 (4.1)	9 (25.7)	10 (28.6)
**Stress intervention and guidance format**				
	Semistandardized stress	191 (22.4)	99 (51.8)	118 (61.8)
	Feedback-on-demand stress	241 (28.3)	95 (39.4)	116 (48.1)
	Self-guided stress	29 (3.4)	8 (27.6)	9 (31.0)

^a^In the mood training, only 1 participant received the feedback-on-demand and 2 self-guided interventions; therefore, adherence was not reported by guidance format for the mood intervention.

There was a significant effect of guidance on treatment adherence (*χ^2^*_2_=49.6; *P*<.001). Planned comparisons of the guidance groups revealed that participants receiving semistandardized guidance (563/851, 66.2%; mean 7, SD 4.8; range 1-25) completed significantly more sessions than participants in the self-guided group (35/851, 4.1%; mean 4.8, SD 4.7; range 1-16; β=−.39; SE 0.08; *z*=−4.93; *P<*.001), participants in the semistandardized guided group completed significantly more sessions than participants in the feedback-on-demand guided group (253/851, 29.7%; mean 6, SD 4.8; range 1-20; β=.16; SE 0.03; *z*=5.26; *P<*.001), and participants in the feedback-on-demand guided group completed significantly more sessions than participants in the self-guided group (β=−.23; SE 0.08; *z*=−2.83; *P=*.005).

There was a significant effect of the baseline depressive symptom severity on treatment adherence (χ^2^_2_=20.9, *P<*.001). Planned comparisons of the guidance groups revealed that participants reporting severe depressive symptoms (327/851, 38.4%; mean 6.9, SD 4.6; range 1-25) completed significantly more sessions than participants reporting mild depressive symptoms (214/851, 25.1%; mean 6, SD 5.1; range 1-24; β=−.15; SE 0.04; *z*=−4.26; *P*<.001), and participants reporting moderate depressive symptoms (310/851, 36.4%; mean 6.8, SD 4.9; range 1-20) completed significantly more sessions than participants reporting mild depressive symptoms (β=.14; SE 0.04; *z*=3.85; *P*<.001). There was no difference between participants reporting moderate and severe symptoms with regard to the number of sessions completed (β=−.01; SE 0.03; *z*=−0.43; *P*=.67).

Additional analyses showed that neither the baseline depressive symptom severity (χ^2^_1_=0.8; *P*=.39), perceived stress (χ^2^_1_=2.3; *P*=.13), nor insomnia severity (χ^2^_1_=0.0; *P*=.83) predicted whether clients started the treatment or not.

#### Participant Satisfaction

Of all participants, 31.8% (271/851) provided data on the CSQ-8. Overall, satisfaction with the intervention, measured using the CSQ-8, was high. Most participants (227/271, 83.8%) indicated that they were satisfied in an overall sense (“very satisfied” or “mostly satisfied”). They rated the training as being of high quality (249/271, 91.9%) and the type of intervention they wanted to receive (223/271, 82.3%). They indicated that the intervention met their needs (264/271, 97.4% “almost all” and “most of them”) and helped them deal effectively with problems (231/271, 85.2%). Moreover, 81.9% (222/271 were satisfied with the amount of help they received and would use the intervention again if they needed to (224/271, 82.7%), and 89.3% (242/271) would recommend it to a friend in need of similar help.

## Discussion

### Principal Findings

This study investigated the use of, effectiveness of, adherence to, and participant satisfaction with 2 iCBT interventions addressing depression- and stress-related symptoms within routinely collected data. A total of 851 participants who reported a PHQ-8 score of >5 and who started the GET.ON Mood Enhancer and GET.ON Stress interventions (today HelloBetter) were included in the reported analysis. Approximately half (461/851, 54.2%) of the participants followed the stress intervention, and of those participants following the stress intervention, 41% (189/461) received semistandardized guidance, 52% (240/461) received feedback-on-demand guidance, and 7% (32/461) followed the self-guided intervention. Of those following the mood intervention, all but 3 patients (349/352, 99.2%) received semistandardized guidance. The results confirm the effect of the service in reducing depressive symptom severity, perceived stress, and insomnia in routine care. Guidance significantly moderated both the effectiveness of and adherence to the interventions regarding reducing depressive symptom severity. Approximately half (150/289, 51.9%) of the participants showed a reliable change in depressive symptom severity after treatment, and 3.8% (11/289) showed reliable symptom deterioration. In such cases, participants were referred to routine health care services. Participant satisfaction was high, and across all patient groups and both interventions, approximately half (405/851, 47.6%) of the participants completed all modules of the intervention provided.

The results indicate the effectiveness of the service in reducing depressive symptom severity, with a large within-group effect size of *d*=−0.92 (ranging from *d*=−1.36 in the group of participants with severe depression to *d*=−0.21 in the group of participants with mild depression). Furthermore, the results indicate the interventions to be effective in reducing perceived stress, with a large effect size of *d*=−1.02 (ranging from *d*=−1.25 in the group of participants with moderate depression to *d*=−0.56 in the group of participants with mild depression), and insomnia severity, with a medium effect size of *d*=−0.75 (ranging from *d*=−1.01 in the group of participants with severe depression to *d*=−0.22 in the group of self-guided participants). The within-group effect sizes for the guided intervention correspond to the effect sizes reported for similar interventions in routine care [65-68]. Furthermore, these results indicate the successful implementation of iCBT interventions in (German) mental health care, where the interventions show similar effects in routine care as in previous RCTs reporting within-group effects of *d*=1.54 [37].

Interestingly, participants receiving the self-guided intervention showed similar symptom reduction as the participants in the semistandardized guidance group. This result might be due to the recommendation procedures based on the screening questions and the resulting differences between the groups. Participants received a recommendation on the treatment and guidance format. Although they could choose to opt out of the semistandardized guidance after it was recommended, most participants (840/851, 98.7%) followed the recommendations regarding treatment and guidance format. People in the self-guided group might have believed that they truly did not need more guidance, and therefore, this guidance format sufficed for them. Participants experiencing high depression-related symptoms, whether combined with high stress-related symptoms, received the semistandardized guided intervention. Less affected patients received the self-guided intervention, and this group could benefit from this low-threshold intervention. This also indicates the usefulness of the GET.ON recommendation scheme. Participants in the feedback-on-demand group showed a lower symptom reduction. Participants receiving feedback-on-demand interventions who were recommended this type showed mild to moderate depressive symptoms. A potential explanation for the lower but still significant symptom reduction of *d*=−0.72 might be that participants in need of more support did not make use of the option to ask questions or request feedback from an online coach. Future studies should analyze this participant group, potentially looking at individual symptom trajectories and routine outcome monitoring, and evaluate the need for additional support of potential subgroups.

In this study, 59.6% (163/273) of participants were first-time help seekers reporting not having received previous psychotherapeutic treatment. This result underlines the great potential of iCBT interventions in reaching populations that otherwise would not seek or find adequate help. In addition, participants in this study were highly educated (503/843, 59.7% had the test level of education). Higher education is associated with a positive view of psychotherapy in general and higher service use in routine care [69,70], and the distribution of educational levels in our sample is comparable with distributions in trials on iCBT for the treatment of depression [13]. The higher proportion of female participants is in line with the use rates of psychotherapy found in epidemiological studies in Germany and elsewhere [71,72]. This effect might be explained by gender differences in help-seeking behavior rather than being related to iCBT service–related factors [73]. Future studies should focus on ways to encourage men to use iCBT interventions. In addition, the mean age of 41.7 years was comparable with the results found in epidemiological studies on the use of face-to-face therapy [74] and with the results of the previous RCT on the efficacy of GET.ON Stress [37].

The high participant satisfaction reported by those who answered the CSQ-8 (ranging between 82% and 97% agreement) is comparable with participant satisfaction rates in similar interventions [75,76]. Although this result must be interpreted with caution as only a third of the participants (271/851, 31.8%) provided information on the CSQ-8, the result is an indicator of the acceptability of iCBT once people have chosen to use such an intervention.

The deterioration rate of 3.8% is distributed across all guidance formats as well as both the stress and mood interventions. Deterioration rates of this size have been identified in other studies [67,75,77] as well as in the results of the previous RCT on the efficacy of GET.ON Stress [37] and other comparable RCTs [78]. Symptom deterioration and other negative effects should be monitored, and appropriate care for participants should be provided. GET.ON has a protocolized system in place where participants showing symptom deterioration are referred to more intensive care. Further phase-IV trials should focus on possible predictors of deterioration, and the investigation of individual patient trajectories should be used to prevent potential deterioration.

Adherence rates in this study were similar to those observed in comparable studies [66,67,79] but lower than those yielded in a randomized, controlled setting [80,81]. Differences between adherence to iCBT interventions in routine care as compared with in RCTs might be due to the absence of the SMS text message coaching part in the stress management intervention, a format that has been proven to be effective in a similar setting [82]. Moreover, an assumed adherence-fostering effect of randomized controlled settings versus routine care might contribute to this difference [83]. Participants might be more likely to stop treatment once they have recovered, even in agreement with their assigned professional.

The effect of guidance on internet-based interventions’ efficacy in reducing depressive symptom severity has also been investigated in other studies, and the first results indicate that guidance has a significant influence on adherence [15,16,80]. Zarski et al [80] found that adherence to GET.ON Stress delivered with feedback-on-demand guidance was equivalent to the intervention including semistandardized guidance within pooled RCT data [80]. In contrast, our data suggest that there was a difference between participants receiving feedback-on-demand or semistandardized guidance. This difference might be due to the recommendation into a specific guidance format and the subsequent fact that guidance was only offered to a specific group of participants. Another influence might be the different research settings—RCT versus routine care. Recently, Baumel et al [84] found that indications for trial settings have an impact on user engagement in self-guided interventions. A possible explanation might be that the feedback-on-demand guidance condition in RCTs still provides additional contacts because of routine study administration. This might have an additional adherence-fostering effect, adding to a difference between feedback-on-demand and semistandardized guidance not present in the routine care application of the service.

### Limitations

Although we observed a significantly greater change in depressive symptom severity in the group of participants receiving semistandardized guidance than in the group receiving no guidance, it must be noted that participants with lower baseline symptom severity were assigned to partake in the feedback-on-demand guided or self-guided intervention, whereas participants with higher baseline symptom severity were assigned the semistandardized guided intervention. This resulted in baseline differences in symptom severity in these groups, and a greater change can be expected in a population with a higher burden. This also accounts for the significant difference between the mood and stress interventions—participants reporting a lower depressive symptom severity were mainly recommended the stress intervention, and participants with higher depressive symptom severity were recommended the mood intervention. Again, part of this difference must be assumed to be due to this baseline difference in the measure. Moreover, the group comparison of mild, moderate, and severe symptom severity should be interpreted with caution as the outcome and predictor are defined by the same measure. In this research setup, a causal relationship between guidance and effectiveness cannot be assumed or interpreted. Moreover, the limited total sample size of this implementation project, the small sample sizes in the different analysis groups, and the implementation in the German public health care context might limit the generalizability of results to people with depression overall. In addition, the absence of postdata poses a constraining impact on the generalizability and reliability of the results of this study. As an additional limitation, it needs to be mentioned that dividing the sample into subsets with regard to guidance and mood resulted in group effect sizes with large CIs. Furthermore, the report of effect sizes might be biased as they do not take the random variance of participants into account.

### Conclusions

This study adds to the body of literature on the effectiveness of iCBT under routine care conditions. Its main strength is that the interventions described have been thoroughly researched and their clinical efficacy has been established. They are now implemented in German mental health care as tested. Data reported are routinely collected and, therefore, depict routine outcomes and adherence without the potential confounding of additional research. Such results are crucial as depression disorders are highly prevalent and costly and are substantially undertreated, and the presented results support the hypothesis that iCBT may well be able to help bridge this gap in reaching people in need of actual routine care.

To overcome some of the limitations of this study, future research should focus on the analysis of individual participant trajectories to identify characteristics of those participants not benefiting from the intervention provided. Furthermore, these findings must be replicated in different settings and samples to gain further knowledge of the influence of such factors on iCBT in routine care. In addition, future studies could investigate further the recommendation processes, such as those applied in the GET.ON (HelloBetter) service, to ensure that the maximum number of participants receive the intervention best suited to their needs.

The results of this early implementation study indicate the effectiveness of semistandardized guided, feedback-on-demand guided, and self-guided iCBT interventions for depression- and stress-related symptoms under routine care conditions. These findings highlight that the provision of semistandardized, feedback-on-demand, and self-guided iCBT interventions is possible in the German mental health context and that such interventions can help reach more people in need of treatment for depressive or stress-related symptoms.

## Appendix

Multimedia Appendix 1 Sensitivity analysis.

## Abbreviations

CSQ-8: Client Satisfaction Questionnaire–8
HIC: health insurance company
iCBT: internet-based cognitive behavioral therapy
ISI: Insomnia Severity Index
PHQ-8: Patient Health Questionnaire–8
PHQ-9: Patient Health Questionnaire–9
PSS-10: Perceived Stress Scale
RCT: randomized controlled trial

## Data Availability

The data used in this research will be made available by the authors upon reasonable request.

## References

[1] (2017). Depression and other common mental disorders: global health estimates. World Health Organization.

[2] Whiteford HA, Degenhardt L, Rehm J, Baxter AJ, Ferrari AJ, Erskine HE, Charlson FJ, Norman RE, Flaxman AD, Johns N, Burstein R, Murray CJ, Vos T (2013). Global burden of disease attributable to mental and substance use disorders: findings from the Global Burden of Disease Study 2010. Lancet.

[3] Kessler RC (2012). The costs of depression. Psychiatr Clin North Am.

[4] Cuijpers P, Karyotaki E, Reijnders M, Ebert DD (2019). Was Eysenck right after all? A reassessment of the effects of psychotherapy for adult depression. Epidemiol Psychiatr Sci.

[5] Thornicroft G, Chatterji S, Evans-Lacko S, Gruber M, Sampson N, Aguilar-Gaxiola S, Al-Hamzawi A, Alonso J, Andrade L, Borges G, Bruffaerts R, Bunting B, de Almeida JM, Florescu S, de Girolamo G, Gureje O, Haro JM, He Y, Hinkov H, Karam E, Kawakami N, Lee S, Navarro-Mateu F, Piazza M, Posada-Villa J, de Galvis YT, Kessler RC (2017). Undertreatment of people with major depressive disorder in 21 countries. Br J Psychiatry.

[6] Mack S, Jacobi F, Gerschler A, Strehle J, Höfler M, Busch MA, Maske UE, Hapke U, Seiffert I, Gaebel W, Zielasek J, Maier W, Wittchen HU (2014). Self-reported utilization of mental health services in the adult German population--evidence for unmet needs? Results of the DEGS1-Mental Health module (DEGS1-MH). Int J Methods Psychiatr Res.

[7] Ebert DD, Van Daele T, Nordgreen T, Karekla M, Compare A, Zarbo C, Brugnera A, Øverland S, Trebbi G, Jensen KL, Kaehlke F, Baumeister H (2018). Internet- and mobile-based psychological interventions: applications, efficacy, and potential for improving mental health. Eur Psychol.

[8] Andersson G, Carlbring P, Titov N, Lindefors N (2019). Internet interventions for adults with anxiety and mood disorders: a narrative umbrella review of recent meta-analyses. Can J Psychiatry.

[9] Ebert DD, Berking M, Cuijpers P, Lehr D, Pörtner M, Baumeister H (2015). Increasing the acceptance of internet-based mental health interventions in primary care patients with depressive symptoms. A randomized controlled trial. J Affect Disord.

[10] Apolinário-Hagen J, Fritsche L, Bierhals C, Salewski C (2018). Improving attitudes toward e-mental health services in the general population via psychoeducational information material: a randomized controlled trial. Internet Interv.

[11] Reins JA, Buntrock C, Zimmermann J, Grund S, Harrer M, Lehr D, Baumeister H, Weisel K, Domhardt M, Imamura K, Kawakami N, Spek V, Nobis S, Snoek F, Cuijpers P, Klein JP, Moritz S, Ebert DD (2021). Efficacy and moderators of internet-based interventions in adults with subthreshold depression: an individual participant data meta-analysis of randomized controlled trials. Psychother Psychosom.

[12] Königbauer J, Letsch J, Doebler P, Ebert DD, Baumeister H (2017). Internet- and mobile-based depression interventions for people with diagnosed depression: a systematic review and meta-analysis. J Affect Disord.

[13] Karyotaki E, Ebert DD, Donkin L, Riper H, Twisk J, Burger S, Rozental A, Lange A, Williams AD, Zarski AC, Geraedts A, van Straten A, Kleiboer A, Meyer B, Ünlü Ince BB, Buntrock C, Lehr D, Snoek FJ, Andrews G, Andersson G, Choi I, Ruwaard J, Klein JP, Newby JM, Schröder J, Laferton JA, Van Bastelaar K, Imamura K, Vernmark K, Boß L, Sheeber LB, Kivi M, Berking M, Titov N, Carlbring P, Johansson R, Kenter R, Perini S, Moritz S, Nobis S, Berger T, Kaldo V, Forsell Y, Lindefors N, Kraepelien M, Björkelund C, Kawakami N, Cuijpers P (2018). Do guided internet-based interventions result in clinically relevant changes for patients with depression? An individual participant data meta-analysis. Clin Psychol Rev.

[14] Karyotaki E, Riper H, Twisk J, Hoogendoorn A, Kleiboer A, Mira A, Mackinnon A, Meyer B, Botella C, Littlewood E, Andersson G, Christensen H, Klein JP, Schröder J, Bretón-López J, Scheider J, Griffiths K, Farrer L, Huibers MJ, Phillips R, Gilbody S, Moritz S, Berger T, Pop V, Spek V, Cuijpers P (2017). Efficacy of self-guided internet-based cognitive behavioral therapy in the treatment of depressive symptoms: a meta-analysis of individual participant data. JAMA Psychiatry.

[15] Baumeister H, Reichler L, Munzinger M, Lin J (2014). The impact of guidance on internet-based mental health interventions — a systematic review. Internet Interv.

[16] Karyotaki E, Efthimiou O, Miguel C, Bermpohl FM, Furukawa TA, Cuijpers P, Riper H, Patel V, Mira A, Gemmil AW, Yeung AS, Lange A, Williams AD, Mackinnon A, Geraedts A, van Straten A, Meyer B, Björkelund C, Knaevelsrud C, Beevers CG, Botella C, Strunk DR, Mohr DC, Ebert DD, Kessler D, Richards D, Littlewood E, Forsell E, Feng F, Wang F, Andersson G, Hadjistavropoulos H, Christensen H, Ezawa ID, Choi I, Rosso IM, Klein JP, Shumake J, Garcia-Campayo J, Milgrom J, Smith J, Montero-Marin J, Newby JM, Bretón-López J, Schneider J, Vernmark K, Bücker L, Sheeber LB, Warmerdam L, Farrer L, Heinrich M, Huibers MJ, Kivi M, Kraepelien M, Forand NR, Pugh N, Lindefors N, Lintvedt O, Zagorscak P, Carlbring P, Phillips R, Johansson R, Kessler RC, Brabyn S, Perini S, Rauch SL, Gilbody S, Moritz S, Berger T, Pop V, Kaldo V, Spek V, Forsell Y, Individual Patient Data Meta-Analyses for Depression (IPDMA-DE) Collaboration (2021). Internet-based cognitive behavioral therapy for depression: a systematic review and individual patient data network meta-analysis. JAMA Psychiatry.

[17] Etzelmueller A, Vis C, Karyotaki E, Baumeister H, Titov N, Berking M, Cuijpers P, Riper H, Ebert DD (2020). Effects of internet-based cognitive behavioral therapy in routine care for adults in treatment for depression and anxiety: systematic review and meta-analysis. J Med Internet Res.

[18] Andrews G, Basu A, Cuijpers P, Craske MG, McEvoy P, English CL, Newby JM (2018). Computer therapy for the anxiety and depression disorders is effective, acceptable and practical health care: an updated meta-analysis. J Anxiety Disord.

[19] Nobis S, Lehr D, Ebert DD, Baumeister H, Snoek F, Riper H, Berking M (2015). Efficacy of a web-based intervention with mobile phone support in treating depressive symptoms in adults with type 1 and type 2 diabetes: a randomized controlled trial. Diabetes Care.

[20] Ebert DD, Nobis S, Lehr D, Baumeister H, Riper H, Auerbach RP, Snoek F, Cuijpers P, Berking M (2017). The 6-month effectiveness of Internet-based guided self-help for depression in adults with Type 1 and 2 diabetes mellitus. Diabet Med.

[21] Reins JA, Boß L, Lehr D, Berking M, Ebert DD (2019). The more I got, the less I need? Efficacy of internet-based guided self-help compared to online psychoeducation for major depressive disorder. J Affect Disord.

[22] Buntrock C, Ebert D, Lehr D, Riper H, Smit F, Cuijpers P, Berking M (2015). Effectiveness of a web-based cognitive behavioural intervention for subthreshold depression: pragmatic randomised controlled trial. Psychother Psychosom.

[23] Ebert DD, Buntrock C, Lehr D, Smit F, Riper H, Baumeister H, Cuijpers P, Berking M (2018). Effectiveness of web- and mobile-based treatment of subthreshold depression with adherence-focused guidance: a single-blind randomized controlled trial. Behav Ther.

[24] Schlicker S, Weisel KK, Buntrock C, Berking M, Nobis S, Lehr D, Baumeister H, Snoek FJ, Riper H, Ebert DD (2019). Do nonsuicidal severely depressed individuals with diabetes profit from internet-based guided self-help? Secondary analyses of a pragmatic randomized trial. J Diabetes Res.

[25] Buntrock C, Ebert DD, Lehr D, Smit F, Riper H, Berking M, Cuijpers P (2016). Effect of a web-based guided self-help intervention for prevention of major depression in adults with subthreshold depression: a randomized clinical trial. JAMA.

[26] Nobis S, Ebert DD, Lehr D, Smit F, Buntrock C, Berking M, Baumeister H, Snoek F, Funk B, Riper H (2018). Web-based intervention for depressive symptoms in adults with types 1 and 2 diabetes mellitus: a health economic evaluation. Br J Psychiatry.

[27] Ebert DD, Kählke F, Buntrock C, Berking M, Smit F, Heber E, Baumeister H, Funk B, Riper H, Lehr D (2018). A health economic outcome evaluation of an internet-based mobile-supported stress management intervention for employees. Scand J Work Environ Health.

[28] Andersson G, Titov N (2014). Advantages and limitations of Internet-based interventions for common mental disorders. World Psychiatry.

[29] (2011). BPtK-Studie Zu Wartezeiten in Der Ambulanten Psychotherapeutischen Versorgung: Umfrage Der Landespsycho-Therapeutenkammern Und Der BPtK. Bundes Psychotherapeuten Kammer.

[30] Andrade LH, Alonso J, Mneimneh Z, Wells JE, Al-Hamzawi A, Borges G, Bromet E, Bruffaerts R, de Girolamo G, de Graaf R, Florescu S, Gureje O, Hinkov HR, Hu C, Huang Y, Hwang I, Jin R, Karam EG, Kovess-Masfety V, Levinson D, Matschinger H, O'Neill S, Posada-Villa J, Sagar R, Sampson NA, Sasu C, Stein DJ, Takeshima T, Viana MC, Xavier M, Kessler RC (2014). Barriers to mental health treatment: results from the WHO World Mental Health surveys. Psychol Med.

[31] Schnyder N, Panczak R, Groth N, Schultze-Lutter F (2017). Association between mental health-related stigma and active help-seeking: systematic review and meta-analysis. Br J Psychiatry.

[32] Ebert DD, Heber E, Berking M, Riper H, Cuijpers P, Funk B, Lehr D (2016). Self-guided internet-based and mobile-based stress management for employees: results of a randomised controlled trial. Occup Environ Med.

[33] Ebert DD, Lehr D, Boß L, Riper H, Cuijpers P, Andersson G, Thiart H, Heber E, Berking M (2014). Efficacy of an internet-based problem-solving training for teachers: results of a randomized controlled trial. Scand J Work Environ Health.

[34] Cuijpers P, Cristea IA, Karyotaki E, Reijnders M, Huibers MJ (2016). How effective are cognitive behavior therapies for major depression and anxiety disorders? A meta-analytic update of the evidence. World Psychiatry.

[35] Butler AC, Chapman JE, Forman EM, Beck AT (2006). The empirical status of cognitive-behavioral therapy: a review of meta-analyses. Clin Psychol Rev.

[36] Weisel KK, Lehr D, Heber E, Zarski AC, Berking M, Riper H, Ebert DD (2018). Severely burdened individuals do not need to be excluded from internet-based and mobile-based stress management: effect modifiers of treatment outcomes from three randomized controlled trials. J Med Internet Res.

[37] Heber E, Lehr D, Ebert DD, Berking M, Riper H (2016). Web-based and mobile stress management intervention for employees: a randomized controlled trial. J Med Internet Res.

[38] Ebert DD, Lehr D, Heber E, Riper H, Cuijpers P, Berking M (2016). Internet- and mobile-based stress management for employees with adherence-focused guidance: efficacy and mechanism of change. Scand J Work Environ Health.

[39] Harrer M, Adam SH, Fleischmann RJ, Baumeister H, Auerbach R, Bruffaerts R, Cuijpers P, Kessler RC, Berking M, Lehr D, Ebert DD (2018). Effectiveness of an internet- and app-based intervention for college students with elevated stress: randomized controlled trial. J Med Internet Res.

[40] Wells KB, Stewart A, Hays RD, Burnam MA, Rogers W, Daniels M, Berry S, Greenfield S, Ware J (1989). The functioning and well-being of depressed patients: results from the medical outcomes study. RAND Corporation.

[41] Rothwell PM (2005). External validity of randomised controlled trials: "to whom do the results of this trial apply?". Lancet.

[42] Singal AG, Higgins PD, Waljee AK (2014). A primer on effectiveness and efficacy trials. Clin Transl Gastroenterol.

[43] Ebert DD, Baumeister H (2017). Internet-based self-help interventions for depression in routine care. JAMA Psychiatry.

[44] van der Lem R, van der Wee NJ, van Veen T, Zitman FG (2012). Efficacy versus effectiveness: a direct comparison of the outcome of treatment for mild to moderate depression in randomized controlled trials and daily practice. Psychother Psychosom.

[45] Flay BR, Biglan A, Boruch RF, Castro FG, Gottfredson D, Kellam S, Mościcki EK, Schinke S, Valentine JC, Ji P (2005). Standards of evidence: criteria for efficacy, effectiveness and dissemination. Prev Sci.

[46] Kroenke K, Spitzer RL (2002). The PHQ-9: a new depression diagnostic and severity measure. Psychiatric Annals.

[47] Kroenke K, Spitzer RL, Williams JB (2001). The PHQ-9: validity of a brief depression severity measure. J Gen Intern Med.

[48] Vis C, Kleiboer A, Prior R, Bønes E, Cavallo M, Clark SA, Dozeman E, Ebert D, Etzelmueller A, Favaretto G, Zabala AF, Kolstrup N, Mancin S, Mathiassen K, Myrbakk VN, Mol M, Jimenez JP, Power K, van Schaik A, Wright C, Zanalda E, Pederson CD, Smit J, Riper H (2015). Implementing and up-scaling evidence-based eMental health in Europe: the study protocol for the MasterMind project. Internet Interv.

[49] Corson K, Gerrity MS, Dobscha SK (2004). Screening for depression and suicidality in a VA primary care setting: 2 items are better than 1 item. Am J Manag Care.

[50] Cohen S, Kamarck T, Mermelstein R (1983). A global measure of perceived stress. J Health Soc Behav.

[51] Reis D, Lehr D, Heber E, Ebert DD (2019). The German version of the Perceived Stress Scale (PSS-10): evaluation of dimensionality, validity, and measurement invariance with exploratory and confirmatory bifactor modeling. Assessment.

[52] Bastien CH, Vallières A, Morin CM (2001). Validation of the insomnia severity index as an outcome measure for insomnia research. Sleep Med.

[53] (Model) Professional Code for Physicians in Germany - MBO-Ä 1997 - The Resolutions of the 121st German Medical Assembly 2018 in Erfurt as amended by a Resolution of the Executive Board of the German Medical Association (English version). German Medical Association.

[54] Lazarus RS, Folkman S (1984). Stress, Appraisal, and Coping.

[55] Heber E, Ebert DD, Lehr D, Nobis S, Berking M, Riper H (2013). Efficacy and cost-effectiveness of a web-based and mobile stress-management intervention for employees: design of a randomized controlled trial. BMC Public Health.

[56] Erbe D, Eichert HC, Rietz C, Ebert D (2016). Interformat reliability of the patient health questionnaire: validation of the computerized version of the PHQ-9. Internet Interv.

[57] Cohen S, Janicki-Deverts D (2012). Who's stressed? Distributions of psychological stress in the United States in probability samples from 1983, 2006, and 2009. J Appl Soc Psychol.

[58] Nguyen TD, Attkisson CC, Stegner BL (1983). Assessment of patient satisfaction: development and refinement of a service evaluation questionnaire. Eval Program Plann.

[59] Boß L, Lehr D, Reis D, Vis C, Riper H, Berking M, Ebert DD (2016). Reliability and validity of assessing user satisfaction with web-based health interventions. J Med Internet Res.

[60] Satterthwaite FE (1946). An approximate distribution of estimates of variance components. Biometrics.

[61] Feingold A (2009). Effect sizes for growth-modeling analysis for controlled clinical trials in the same metric as for classical analysis. Psychol Methods.

[62] Armstrong RA (2014). When to use the Bonferroni correction. Ophthalmic Physiol Opt.

[63] Jacobson NS, Truax P (1991). Clinical significance: a statistical approach to defining meaningful change in psychotherapy research. J Consult Clin Psychol.

[64] R Core Team R: a language and environment for statistical computing. R Foundation for Statistical Computing.

[65] Hedman E, Ljótsson B, Rück C, Bergström J, Andersson G, Kaldo V, Jansson L, Andersson E, Andersson E, Blom K, El Alaoui S, Falk L, Ivarsson J, Nasri B, Rydh S, Lindefors N (2013). Effectiveness of internet-based cognitive behaviour therapy for panic disorder in routine psychiatric care. Acta Psychiatr Scand.

[66] Mathiasen K, Riper H, Andersen TE, Roessler KK (2018). Guided internet-based cognitive behavioral therapy for adult depression and anxiety in routine secondary care: observational study. J Med Internet Res.

[67] Ruwaard J, Lange A, Schrieken B, Dolan CV, Emmelkamp P (2012). The effectiveness of online cognitive behavioral treatment in routine clinical practice. PLoS One.

[68] Morrison C, Walker G, Ruggeri K, Hacker Hughes JH (2014). An implementation pilot of the MindBalance web-based intervention for depression in three IAPT services. Cogn Behav Ther.

[69] Petrowski K, Hessel A, Körner A, Weidner K, Brähler E, Hinz A (2014). Die Einstellung zur Psychotherapie in der Allgemeinbevölkerung. Psychother Psych Med.

[70] Gallas C, Kächele H, Kraft S, Kordy H, Puschner B (2008). Inanspruchnahme, verlauf und ergebnis ambulanter psychotherapie: Befunde der TRANS-OP-studie und deren implikationen für die richtlinienpsychotherapie. Psychotherapeut.

[71] Albani C, Blaser G, Geyer M, Schmutzer G, Goldschmidt S, Brähler E (2009). Wer nimmt in Deutschland ambulante Psychotherapie in Anspruch?. Psychother Psychosom Med Psychol.

[72] Seidler ZE, Dawes AJ, Rice SM, Oliffe JL, Dhillon HM (2016). The role of masculinity in men's help-seeking for depression: a systematic review. Clin Psychol Rev.

[73] Thompson AE, Anisimowicz Y, Miedema B, Hogg W, Wodchis WP, Aubrey-Bassler K (2016). The influence of gender and other patient characteristics on health care-seeking behaviour: a QUALICOPC study. BMC Fam Pract.

[74] Epping J, Muschik D, Geyer S (2017). Social inequalities in the utilization of outpatient psychotherapy: analyses of registry data from German statutory health insurance. Int J Equity Health.

[75] Titov N, Dear BF, Staples LG, Bennett-Levy J, Klein B, Rapee RM, Andersson G, Purtell C, Bezuidenhout G, Nielssen OB (2017). The first 30 months of the MindSpot clinic: evaluation of a national e-mental health service against project objectives. Aust N Z J Psychiatry.

[76] Etzelmueller A, Radkovsky A, Hannig W, Berking M, Ebert DD (2018). Patient's experience with blended video- and internet based cognitive behavioural therapy service in routine care. Internet Interv.

[77] Hadjistavropoulos HD, Nugent MM, Alberts NM, Staples L, Dear BF, Titov N (2016). Transdiagnostic internet-delivered cognitive behaviour therapy in Canada: an open trial comparing results of a specialized online clinic and nonspecialized community clinics. J Anxiety Disord.

[78] Rozental A, Magnusson K, Boettcher J, Andersson G, Carlbring P (2017). For better or worse: an individual patient data meta-analysis of deterioration among participants receiving internet-based cognitive behavior therapy. J Consult Clin Psychol.

[79] Hedman E, Ljótsson B, Kaldo V, Hesser H, El Alaoui S, Kraepelien M, Andersson E, Rück C, Svanborg C, Andersson G, Lindefors N (2014). Effectiveness of internet-based cognitive behaviour therapy for depression in routine psychiatric care. J Affect Disord.

[80] Zarski AC, Lehr D, Berking M, Riper H, Cuijpers P, Ebert DD (2016). Adherence to internet-based mobile-supported stress management: a pooled analysis of individual participant data from three randomized controlled trials. J Med Internet Res.

[81] van Ballegooijen W, Cuijpers P, van Straten A, Karyotaki E, Andersson G, Smit JH, Riper H (2014). Adherence to Internet-based and face-to-face cognitive behavioural therapy for depression: a meta-analysis. PLoS One.

[82] Eckert M, Ebert DD, Lehr D, Sieland B, Berking M (2018). Does SMS-support make a difference? Effectiveness of a two-week online-training to overcome procrastination. A randomized controlled trial. Front Psychol.

[83] Ebert DD, Baumeister H (2016). nternet- und mobil-basierte Interventionen in der Psychotherapie: Ein Überblick. Psychotherapeutenjournal.

[84] Baumel A, Edan S, Kane JM (2019). Is there a trial bias impacting user engagement with unguided e-mental health interventions? A systematic comparison of published reports and real-world usage of the same programs. Transl Behav Med.

