# Supplementary material for: The Evaluation of the GET.ON Nationwide Web-Only Treatment Service for Depression- and Stress-Related Symptoms: Naturalistic Trial
Source: J Med Internet Res. 2024 Feb 1;26:e42976. doi: 10.2196/42976 (PMC10870208; doi:10.2196/42976)
Supplement: Multimedia Appendix 1 [file jmir_v26i1e42976_app1.docx]

*Appendix A. Sensitivity analysis*

*1. Study completer subset – linear mixed-effects model outcomes and effect sizes for depressive symptom severity (measured by the PHQ-8 questionnaire).*

|  | Estimate | SD | t | F | df | p | Effect size | 95%-CI (min) | 95%-CI (max) |
| --- | --- | --- | --- | --- | --- | --- | --- | --- | --- |
| *All starters (n = 851)* |  |  |  | 262.17 | 289.25 | < .001 |  |  |  |
| *Time* | -4.95 | 0.31 | -16.19 |  | 289.25 | < .001 | -0.98 | -1.39 | -0.57 |
| *Guidance* |  |  |  | 27.76 | 286.64 | < .001 |  |  |  |
| *Semi-standardized guidance* | -6.22 | 0.35 | -17.84 |  |  | < .001 | -1.20 | -1.55 | -0.85 |
| *Feedback-on-demand guidance* | -1.59 | 0.48 | -3.34 |  |  | .001 | -0.39 | -0.87 | 0.09 |
| *Self-guided* | -0.71 | 0.36 | -1.99 |  |  | < .001 | -0.78 | -1.12 | -0.44 |
| *Guidance: Semi-stand. x Self-guided* | 5.50 | 1.83 | 3.00 |  |  | .003 |  |  |  |
| *Guidance: Semi-stand. x Adherence* | 4.63 | 0.66 | 7.06 |  |  | < .001 |  |  |  |
| *Guidance: FoD x Self-guided* | 0.88 | 1.89 | 0.46 |  |  | .64 |  |  |  |
| *Intervention* |  |  |  | 46.36 | 273.00 | < .001 |  |  |  |
| *Mood* | -7.14 | 0.46 | -15.68 |  |  | < .001 | -1.31 | -1.78 | -0.84 |
| *Stress* | -3.32 | 0.34 | -9.66 |  |  | < .001 | -0.85 | -1.17 | -0.53 |
| *Symptom Severity* |  |  |  | 44.68 | 301.30 | < .001 |  |  |  |
| *Severe* | -7.52 | 0.46 | -16.45 |  |  | < .001 | -1.37 | -1.85 | -0.89 |
| *Medium* | -4.18 | 0.42 | -9.86 |  |  | < .001 | -0.99 | -1.39 | -0.59 |
| *Mild* | -0.93 | 0.46 | -2.03 |  |  | .05 | -0.23 | -0.75 | 0.29 |
| *Symptom Severity: Mild x Severe* | -6.58 | 0.72 | -9.19 |  |  | < .001 |  |  |  |
| *Symptom Severity: Mild x Medium* | -3.25 | 0.74 | -4.41 |  |  | < .001 |  |  |  |
| *Symptom Severity: Medium x Severe* | 3.34 | 0.60 | 5.57 |  |  | < .001 |  |  |  |

SD = Standard deviation, df = degrees of freedom, CI = confidence interval.

*2. Treatment completer subset – linear mixed-effects model outcomes and effect sizes for depressive symptom severity (measured by the PHQ-8 questionnaire).*

|  | *Estimate* | *SD* | *t* | *F* | *df* | *p* | *Effect size* | *95%-CI (min)* | *95%-CI (max)* |
| --- | --- | --- | --- | --- | --- | --- | --- | --- | --- |
| *All starters (n = 851)* |  |  |  | 297.80 | 328.07 | < .001 |  |  |  |
| *Time* | -5.20 | 0.30 | -17.26 |  | 328.07 | < .001 | -1.09 | -1.45 | -0.73 |
| *Guidance* |  |  |  | 31.34 | 376.66 | < .001 |  |  |  |
| *Semi-standardized guidance* | -6.47 | 0.33 | -19.78 |  |  | < .001 | -1.32 | -1.63 | -1.01 |
| *Feedback-on-demand guidance* | -1.68 | 0.41 | -4.04 |  |  | < .001 | -0.43 | -0.87 | 0.01 |
| *Self-guided* | -0.91 | 0.50 | -1.81 |  |  | .15 | -0.88 | -1.26 | -0.50 |
| *Guidance: Semi-stand. x Self-guided* | 5.18 | 1.88 | 2.76 |  |  | .006 |  |  |  |
| *Guidance: Semi-stand. x Adherence* | 4.78 | 0.63 | 7.62 |  |  | < .001 |  |  |  |
| *Guidance: FoD x Self-guided* | 0.40 | 1.93 | 0.20 |  |  | .84 |  |  |  |
| *Intervention* |  |  |  | 59.57 | 358.93 | < .001 |  |  |  |
| *Mood* | -7.36 | 0.40 | -18.28 |  |  | < .001 | -1.46 | -1.86 | -1.06 |
| *Stress* | -3.42 | 0.32 | -10.64 |  |  | < .001 | -0.90 | -1.19 | -0.61 |
| *Symptom Severity* |  |  |  | 63.28 | 420.68 | < .001 |  |  |  |
| *Severe* | -7.85 | 0.40 | -19.77 |  |  | < .001 | -1.55 | -1.96 | -1.14 |
| *Medium* | -4.40 | 0.36 | -12.40 |  |  | < .001 | -1.08 | -1.44 | -0.72 |
| *Mild* | -0.84 | 0.45 | -1.85 |  |  | .07 | -0.19 | -0.72 | 0.34 |
| *Symptom Severity: Mild x Severe* | -7.01 | 0.64 | -10.93 |  |  | < .001 |  |  |  |
| *Symptom Severity: Mild x Medium* | -3.57 | 0.66 | -5.42 |  |  | < .001 |  |  |  |
| *Symptom Severity: Medium x Severe* | 3.44 | 0.52 | 6.58 |  |  | < .001 |  |  |  |

SD = Standard deviation, df = degrees of freedom, CI = confidence interval.

*3. Study completer subset – linear mixed-effects model outcomes and effect sizes for stress related symptoms.*

|  | *Estimate* | *SD* | *t* | *F* | *df* | *p* | *Effect size* | *95%-CI (min)* | *95%-CI (max)* |
| --- | --- | --- | --- | --- | --- | --- | --- | --- | --- |
| *All starters (n = 851)* |  |  |  | 317.34 | 289.34 | < .001 |  |  |  |
| *Time* | -8.10 | 0.45 | -17.81 |  | 289.34 | < .001 | -1.08 | -1.70 | -0.46 |
| *Guidance* |  |  |  | 4.54 | 285.77 | .010 |  |  |  |
| *Semi-standardized guidance* | -8.93 | 0.53 | -16.94 |  |  | < .001 | -1.22 | -1.72 | -0.72 |
| *Feedback-on-demand guidance* | -5.93 | 0.93 | -6.39 |  |  | < .001 | -0.75 | -1.67 | 0.17 |
| Self-guided | -5.43 | 1.74 | -3.11 |  |  | .02 | -1.17 | -2.89 | 0.55 |
| Guidance: Semi-stand. x Self-guided | 3.50 | 2.93 | 1.19 |  |  | .23 |  |  |  |
| Guidance: Semi-stand. x Adherence | 3.00 | 1.05 | 2.86 |  |  | .005 |  |  |  |
| *Guidance: FoD x Self-guided* | 0.50 | 3.02 | 0.17 |  |  | .87 |  |  |  |
| *Intervention* |  |  |  | 0.21 | 273.05 | .65 |  |  |  |
| *Mood* | -8.46 | 0.67 | -12.70 |  |  | < .001 | -1.13 | -1.78 | -0.48 |
| *Stress* | -8.05 | 0.63 | -12.73 |  |  | < .001 | -1.12 | -1.70 | -0.54 |
| *Symptom Severity* |  |  |  | 94.49 | 858.05 | < .001 |  |  |  |
| *Severe* | -9.16 | 0.68 | -13.53 |  |  | < .001 | -1.21 | -1.88 | -0.54 |
| *Medium* | -8.53 | 0.71 | -11.98 |  |  | < .001 | -1.28 | -1.91 | -0.65 |
| *Mild* | -5.14 | 1.06 | -4.86 |  |  | < .001 | -0.63 | -1.67 | 0.41 |
| *Symptom Severity: Mild x Severe* | -4.03 | 1.20 | -3.36 |  |  | .001 |  |  |  |
| *Symptom Severity: Mild x Medium* | -3.39 | 1.23 | -2.76 |  |  | .006 |  |  |  |
| *Symptom Severity: Medium x Severe* | 0.63 | 1.00 | 0.63 |  |  | .53 |  |  |  |

SD = Standard deviation, df = degrees of freedom, CI = confidence interval.

*4. Treatment completer subset – linear mixed-effects model outcomes and effect sizes stress related symptoms.*

|  | *Estimate* | *SD* | *t* | *F* | *df* | *p* | *Effect size* | *95%-CI (min)* | *95%-CI (max)* |
| --- | --- | --- | --- | --- | --- | --- | --- | --- | --- |
| *All starters (n = 851)* |  |  |  | 346.05 | 355.22 | < .001 |  |  |  |
| *Time* | -8.25 | 0.44 | -18.60 |  | 355.22 | < .001 | -1.10 | -1.67 | -0.73 |
| *Guidance* |  |  |  | 5.01 | 370.58 | .010 |  |  |  |
| *Semi-standardized guidance* | -9.08 | 0.50 | -18.26 |  |  | < .001 | -1.24 | -1.70 | -1.01 |
| *Feedback-on-demand guidance* | -5.99 | 0.90 | -6.63 |  |  | < .001 | -0.78 | -1.63 | 0.01 |
| Self-guided | -5.54 | 2.08 | -2.67 |  |  | .03 | -1.14 | -3.01 | -0.50 |
| Guidance: Semi-stand. x Self-guided | 3.52 | 3.09 | 1.14 |  |  | .26 |  |  |  |
| Guidance: Semi-stand. x Adherence | 3.09 | 1.02 | 3.03 |  |  | .003 |  |  |  |
| Guidance: FoD x Self-guided | 0.43 | 3.18 | 0.14 |  |  | .90 |  |  |  |
| *Intervention* |  |  |  | 0.08 | 346.40 | .77 |  |  |  |
| *Mood* | -8.55 | 0.62 | -13.69 |  |  | < .001 | -1.17 | -1.76 | -1.06 |
| *Stress* | -8.27 | 0.62 | -13.42 |  |  | < .001 | -1.16 | -1.70 | -0.61 |
| *Symptom Severity* |  |  |  | 94.49 | 858.05 | < .001 |  |  |  |
| *Severe* | -9.31 | 0.62 | -14.94 |  |  | < .001 | -1.26 | -1.87 | -1.14 |
| *Medium* | -8.78 | 0.67 | -13.10 |  |  | < .001 | -1.34 | -1.93 | -0.72 |
| *Mild* | -4.91 | 1.06 | -4.63 |  |  | < .001 | -0.58 | -1.61 | 0.34 |
| *Symptom Severity: Mild x Severe* | -4.40 | 1.15 | -3.81 |  |  | < .001 |  |  |  |
| *Symptom Severity: Mild x Medium* | -3.87 | 1.18 | -3.28 |  |  | .001 |  |  |  |
| *Symptom Severity: Medium x Severe* | 0.52 | 0.94 | 0.56 |  |  | .58 |  |  |  |

SD = Standard deviation, df = degrees of freedom, CI = confidence interval.
